# Metabolomic Analysis of Skeletal Muscle in Aged Mice

**DOI:** 10.1038/s41598-019-46929-8

**Published:** 2019-07-18

**Authors:** Ran Uchitomi, Yukino Hatazawa, Nanami Senoo, Kiyoshi Yoshioka, Mariko Fujita, Takahiko Shimizu, Shinji Miura, Yusuke Ono, Yasutomi Kamei

**Affiliations:** 1 Graduate School of Life and Environmental Sciences, Kyoto Prefectural University, Kyoto, Japan; 2 Laboratories of Nutritional Biochemistry, Graduate School of Nutritional and Environmental Sciences, University of Shizuoka, Shizuoka, Japan; 3 Institute of Molecular Embryology and Genetics, Kumamoto University, Kumamoto, Japan; 4 Department of Endocrinology, Hematology, and Geriatrics, Chiba University Graduate School of Medicine, Chiba, Japan; 5 Aging Stress Response Research Project Team, National Center for Geriatrics and Gerontology, Aichi, Japan

**Keywords:** Ageing, Geriatrics

## Abstract

Sarcopenia is the age-induced, progressive loss of skeletal muscle mass and function. To better understand changes in skeletal muscle during sarcopenia, we performed a metabolomic analysis of skeletal muscle in young (8-week-old) and aged (28-month-old) mice by using capillary electrophoresis with electrospray ionization time-of-flight mass spectrometry. Principal component analysis showed clear changes in metabolites between young and aged mice. Glucose metabolism products were decreased in aged mice, specifically fructose 1,6-diphosphate (0.4-fold) and dihydroxyacetone phosphate (0.6-fold), possibly from decreased glycolytic muscle fibers. Multiple metabolic products associated with phospholipid metabolism were significantly changed in aged mice, which may reflect changes in cell membrane phospholipids of skeletal muscle. Products of polyamine metabolism, which are known to increase nucleic acid and protein synthesis, decreased in spermine (0.5-fold) and spermidine (0.6-fold) levels. By contrast, neurotransmitter levels were increased in skeletal muscle of aged mice, including acetylcholine (1.8-fold), histamine (2.6-fold), and serotonin (1.7-fold). The increase in acetylcholine might compensate for age-associated dropout of neuromuscular junctions, whereas the increases in histamine and serotonin might be due to muscle injury associated with aging. Further analysis focusing on the altered metabolites observed in this study will provide essential data for understanding aging muscles.

## Introduction

Skeletal muscle is the largest organ in the human body, accounting for about 40% of body weight, and it plays important roles in exercise and energy expenditure. Sarcopenia refers to the age-induced, progressive loss of skeletal muscle mass and function, which is accompanied by reduced muscle performance. Individuals with sarcopenia often become bedridden or dependent on a wheelchair, leading to decreased quality of life. Epidemiological studies have shown that people with a high quantity of skeletal muscle and a fast walking speed have greater longevity^1^. Considering the aging populations in developed countries, preventive methods and/or a cure for sarcopenia are needed.

Sarcopenia has been extensively studied using mouse models. Mice have a lifespan of 2***–***3 years. We previously reported a decrease in body weight in C57BL/6 mice older than 20 months^2^. Other studies have reported that a sarcopenia phenotype is observed at age 24 months in C57BL/6 mice^3^. In aged sarcopenia model mice, fast glycolytic muscle fibers are the first to decrease^4,5^ and remodeling of skeletal muscle components, such as decreased collagen gene expression, has also been reported^6^. Phospholipid composition in skeletal muscle is also known to change during atrophy^7^. In aged mice, it has been shown that proliferation and differentiation of muscle satellite cells (precursor cells) are suppressed, resulting in delayed regeneration after muscle injury^8^. Moreover, motor neurons in skeletal muscle are apt to drop out during aging, causing worsening of movement performance^9,10^.

To better understand changes in skeletal muscle during sarcopenia, we conducted a metabolomic analysis of skeletal muscle in young (8-week-old) and aged (28-month-old) mice using capillary electrophoresis with electrospray ionization time-of-flight mass spectrometry (CE-TOFMS). Gene expression analysis was also performed.

## Results and Discussion

**Figure 1:**
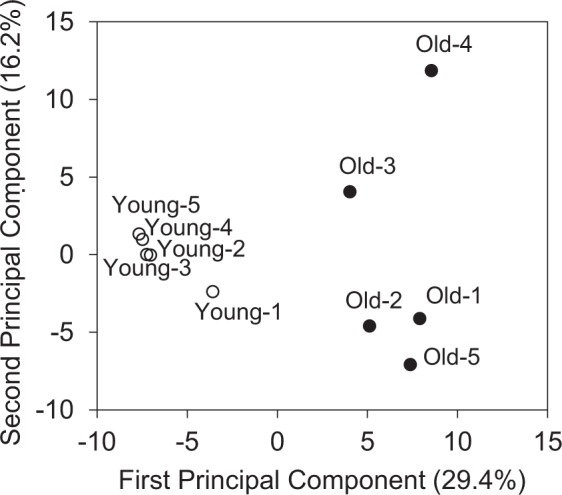
Principal component analysis (PCA) of metabolomic datasets of skeletal muscle from young and aged mice. Five mice were used in each group (Young-1 to Young-5 for young mice and Old-1 to Old-5 for aged mice). PCA was conducted with the determined data peaks using SampleStat ver. 3.14. Plots of young (open circles) and aged mice (filled circles) are clearly distinguished on the first principal component axis (x-axis).

**Figure 2:**
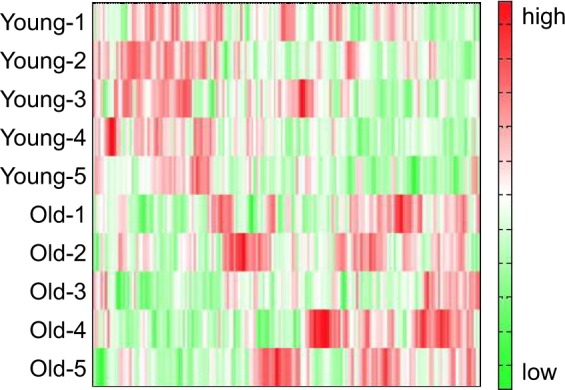
A heat map comparing metabolite changes between young mice and aged mice. The vertical axis shows sample names corresponding to the samples used in Fig. 1 (Young-1 to Young-5 for young mice and Old-1 to Old-5 for aged mice). The heat map patterns between young (upper five lanes) and aged (lower five lanes) are clearly distinguishable. Red indicates that the relative content of metabolites is high, whereas green indicates that the relative content of metabolites is low.

The average body weight was 25.4 ± 0.2 g for young mice and 32.0 ± 0.2 g for aged mice. The average muscle (gastrocnemius) weight was 148.9 ± 3.5 mg for young and 125.4 ± 1.7 mg for aged mice. Adipose tissue weight was 256.5 ± 12.5 mg for young mice and 364.0 ± 46.4 mg for aged mice. Consistent with previous reports^3^, the weight of gastrocnemius muscle in aged mice was significantly lower than that in young mice. In the metabolomic analysis, 176 peaks (119 cations and 57 anions) were detected by the cation and anion modes of CE-TOFMS. The principal component analysis (PCA) results for the detected peaks are shown in Fig. 1. The first principal component effectively and distinctly separated the two groups (x-axis), suggesting that aging of skeletal muscle caused a significant change in the overall metabolite profile of muscle. As demonstrated by the heat map analysis (Fig. 2), skeletal muscle specimens from young and aged mice segregated into two groups, indicating that aging has profound effects on the metabolite profile of the skeletal muscle. Taken together, the PCA and heat map results support that aging significantly influenced the metabolite profile of skeletal muscle and resulted in clear separation of the two groups. The relative area values of the detected metabolic products in young and aged mice are listed in order in Supplementary Table 1. Of these, the metabolites that showed significant changes are listed in Table 1. The numbers of metabolites that showed significant decreases and increases in aged mice relative to young mice were 16 and 24, respectively. These metabolites are involved in various pathways related to glucose, phospholipids, polyamines, neurotransmitters, and amino acids. In the following subsections, the results of the metabolomic analysis are discussed in detail.

**Table 1:** List of significant changes in metabolites in aged mice.

Compound name	Old vs Young		
	Ratio	*p*-value	
Carboxymethyllysine	5.0	0.001	**
1-Methylhistamine	5.0	7.8E-04	***
Histamine	2.6	2.6E-04	***
Diethanolamine	2.5	0.031	*
Pantothenic acid	1.9	0.008	**
Thiamine	1.9	0.002	**
Homocarnosine	1.9	9.6E-05	***
Acetylcholine	1.8	0.001	**
Guanosine	1.8	0.034	*
Serotonin	1.7	0.012	*
Cysteine glutathione disulfide	1.7	0.019	*
*myo*-Inositol 1-phosphate *myo*-Inositol 3-phosphate	1.6	1.2E-04	***
Inosine	1.5	0.022	*
Glycerophosphocholine	1.4	0.012	*
Stachydrine	1.4	0.021	*
*S*-Adenosylmethionine	1.3	0.002	**
Citric acid	1.3	0.005	**
Phe	1.3	0.047	*
Threonic acid	1.3	0.033	*
Isethionic acid	1.3	0.002	**
Gln	1.2	0.011	*
Ethanolamine phosphate	1.2	0.043	*
CMP-*N*-acetylneuraminate	1.2	0.021	*
Taurine	1.1	0.031	*
ATP	0.9	0.003	**
Ala	0.8	0.031	*
Glycerol 3-phosphate	0.8	0.009	**
UTP	0.8	0.002	**
NAD^+^	0.8	1.1E-05	***
Gly	0.7	0.008	**
Sarcosine	0.7	0.002	**
Phosphorylcholine	0.6	0.006	**
β-Ala	0.6	0.002	**
1-Methylnicotinamide	0.6	0.009	**
Spermidine	0.6	0.010	*
Dihydroxyacetone phosphate	0.6	0.033	*
Spermine	0.5	0.001	**
*N*^6^-Methyllysine	0.5	0.001	**
Fructose 1,6-diphosphate	0.4	0.030	*
Hydroxyproline	0.3	4.2E-06	***

“Ratio” is the comparative value of the relative areas (old vs. young). The p-value was calculated using Welch’s *t-*test (***p < 0.001, **p < 0.01, *p < 0.05).

### Glucose metabolism

**Figure 3:**
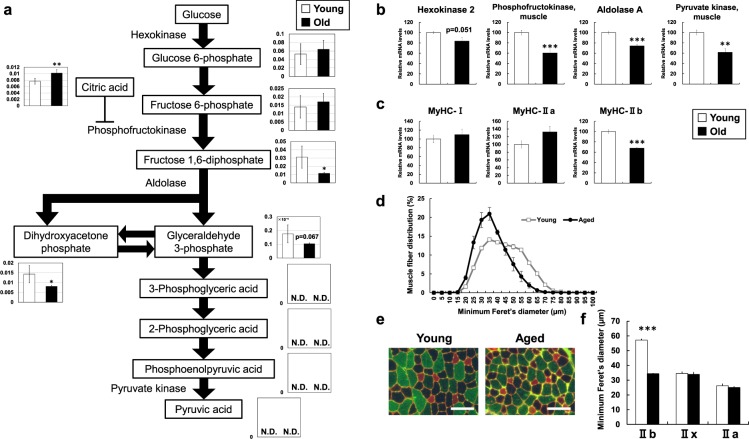
Metabolic changes related to glucose metabolism. (**a**) Metabolite changes in the skeletal muscle of young and aged mice are shown. Relative metabolite changes shown in the graphs were obtained by CE-TOFMS (Supplementary Table 1). Open bars, young mice; filled bars, aged mice. Data are expressed as mean ± SD (N = 5); **p < 0.01, *p < 0.05. Gene expression of (**b**) glucose metabolism and (**c**) myosin heavy chain in skeletal muscle from young and aged mice. Open bars, young mice; filled bars, aged mice. Data are expressed as mean ± SE (N = 5); ***p < 0.001, **p < 0.01. (**d**) Feret’s diameter of total fiber in tibilias anterior (TA) muscle. (**e**) Immunohistochemical analysis of the fiber-type composition in TA muscles. Red, type IIa and laminin; unstained, type IIx; green, type IIb. Scale bars 100 μm. (**f**) Myofiber Feret’s diameter of individual fiber types in TA muscle. ***p < 0.001. There were few type I fibers in all samples.

In the glycolysis pathway, levels of fructose 1,6-diphosphate (0.4-fold), dihydroxyacetone phosphate (0.6-fold), and glyceraldehyde 3-phosphate (0.6-fold) were decreased in aged mice compared with those in young mice (Fig. 3a). The reaction from fructose 6-phosphate to fructose 1,6-diphosphate is catalyzed by phosphofructokinase, a rate-limiting enzyme of glycolysis, which showed decreased gene expression (0.6-fold) by quantitative real-time RT-PCR (Fig. 3b). The enzymatic activity of phosphofructokinase is known to be allosterically inhibited by citric acid^11^. Interestingly, the citric acid level was significantly higher in aged mice (Fig. 3a), suggesting suppressed enzymatic activity of phosphofructokinase. In addition, aldolase mRNA level was lower in aged mice (0.7-fold) than in young mice, which is consistent with lower levels of dihydroxyacetone phosphate and glyceraldehyde 3-phosphate. In the glycolytic pathway, hexokinase and pyruvate kinase are also rate-limiting enzymes and the mRNA levels were 0.8-fold and 0.6-fold lower in aged mice than young mice (Fig. 3b). In our microarray data, we also observed decreased expression of genes encoding other glycolytic pathway enzymes (Supplementary Table 2). Downstream metabolites, such as 3-phosphoglyceric acid, 2-phosphoglyceric acid, phosphoenolpyruvic acid, and pyruvic acid, were unfortunately below the detection level in this study. Overall, the glycolytic pathway appeared to decrease in the skeletal muscle of aged mice.

In skeletal muscle in sarcopenia, fast/white/type IIb glycolytic fiber muscles are reported to preferentially decrease relative to slow/red/type I/type IIa oxidative fiber muscles^4,5^. We observed a decreased mRNA level of myosin heavy chain IIb (MyHC-IIb), which is a fast/white fiber/type IIb (0.7-fold), but not MyHC-I or MyHC-IIa, which are slow/red/type I/type IIa, in aged mice compared with young mice (Fig. 3c). The cross-sectional area (CSA), measured as Feret’s diameter^12^, of dissected muscle fibers was smaller in aged mice than in young mice (Fig. 3d). Immunohistochemistry showed that the CSA of type IIb fibers was significantly smaller in the skeletal muscle of aged mice than in those of young mice, but no significant differences were observed with respect to CSA for IIx and IIa fibers between young and aged mice (Fig. 3e,f). The decrease in glucose metabolites observed in this study may reflect decreased glycolysis in aged mice because of decreased fast/white/type IIb glycolytic fiber muscles in sarcopenia.

### Phospholipids

**Figure 4:**
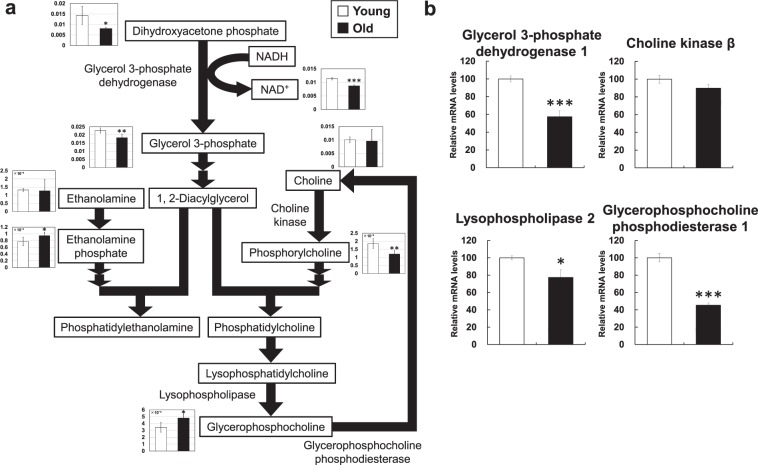
Metabolic changes related to phospholipid metabolism. (**a**) Metabolite changes in the skeletal muscle of young and aged mice are shown. Relative metabolite changes shown in the graphs were obtained by CE-TOFMS (Supplementary Table 1). Open bars, young mice; filled bars, aged mice. Data are expressed as mean ± SD (N = 5); ***p < 0.001, **p < 0.01, *p < 0.05. (**b**) Gene expression of phospholipid metabolism in skeletal muscle from young and aged mice. Open bars, young mice; filled bars, aged mice. Data are expressed as mean ± SE (N = 5); ***p < 0.001, *p < 0.05.

Concerning lipid metabolism, the glycerol 3-phosphate level was significantly decreased (0.8-fold) in aged mice. Glycerol 3-phosphate is used for glycerophospholipids and triglycerides; however, as CE-TOFMS can only detect water-soluble metabolites, but not lipid-soluble metabolites, glycerophospholipids and triglycerides could not be analyzed. Changes in the levels of other glycophospholipid-related metabolites, such as ethanolamine phosphate (1.2-fold increase), phosphorylcholine (0.6-fold decrease), and glycerophosphocholine (1.4-fold increase), were observed (Fig. 4a).

Glycerol 3-phosphate dehydrogenase catalyzes dihydroxyacetone phosphate to glycerol 3-phosphate. In this reaction, NADH is used and NAD^+^ is produced. The glycerol 3-phosphate dehydrogenase mRNA level was decreased (0.6-fold) in aged mice (Fig. 4b), as was the NAD^+^ level (0.8-fold) (Fig. 4a).

Glycerol 3-phosphate is metabolized to 1,2-diacylglycerol. Ethanolamine phosphate (1.2-fold increase) and phosphorylcholine (0.6-fold decrease) are metabolized and bind to 1,2-diacylglycerol to produce phosphatidylethanolamine (PE) and phosphatidylcholine (PC), respectively. They are components of the cell membrane^7^. Glycerophosphocholine (1.4-fold), an intermediate metabolite of the degradation pathway of PC, was increased in aged mice. The changes in metabolite levels related to phospholipid metabolism may reflect changes in the components of cellular phospholipids in the skeletal muscle of aged mice.

Next, the phospholipid composition in skeletal muscle was analyzed and compared between young and aged mice. CE-TOFMS analysis is useful for the comprehensive analysis of water-soluble metabolites in cells, although it cannot detect lipid-soluble metabolites. Thus, we used liquid chromatography-mass spectrometry (LC-MS) analysis to detect phospholipids. PCA analysis of PC and PE clearly showed differential patterns between young and aged mice (Supplementary Fig. 1). Lipid species in the phospholipid fraction (PC and PE) from young and aged mice are shown in Table 2. The level of several PC and PE species differed significantly between young and aged mice. Thus, the suggested phospholipid changes based on CE-TOFMS analysis (Fig. 4) were confirmed by LC-MS analysis.

**Table 2:** Lipid species in the phospholipid fraction (PC and PE) from young and aged mice.

Phospholipid species	Mean		Old vs Young		
	Young	Old	Ratio	*p*-value	
PC (16:0/16:0)	13.62	11.44	0.84	0.004	***
PC (16:0/16:1)	3.26	3.61	1.11	0.107	
PC (16:0/18:1)	9.85	12.48	1.27	8.E-05	***
PC (18:1/18:1)	1.78	2.55	1.43	1.E-04	***
PC (16:0/18:2)	12.01	13.12	1.09	0.252	
PC (16:1/18:2)	1.21	1.63	1.35	0.029	*
PC (18:0/18:2)	3.07	3.98	1.30	0.002	**
PC (18:1/18:2)	2.72	3.77	1.39	0.018	*
PC (18:2/18:2)	2.84	3.91	1.38	0.047	*
PC (16:0/20:4)	9.82	6.70	0.68	3.E-04	***
PC (18:0/20:4)	2.14	1.88	0.88	0.089	
PC (18:1/20:4)	1.14	1.14	0.99	0.943	
PC (18:2/20:4)	1.45	1.26	0.87	0.053	
PC (16:0/22:6)	19.74	16.99	0.86	0.026	*
PC (16:1/22:6)	3.19	3.49	1.09	0.115	
PC (18:0/22:6)	3.85	3.43	0.89	0.200	
PC (18:1/22:6)	1.13	1.50	1.32	0.008	**
PC (18:2/22:6)	2.99	2.74	0.92	0.337	
PE (16:0/18:1)	0.97	1.86	1.92	2.E-04	***
PE (18:0/18:1)	1.53	2.09	1.36	0.231	
PE (18:1/18:1)	3.26	5.73	1.76	0.020	*
PE (16:0/18:2)	1.99	3.11	1.57	0.003	**
PE (18:0/18:2)	8.33	10.13	1.22	0.039	*
PE (18:1/18:2)	2.19	4.26	1.95	2.E-05	***
PE (18:0/20:4)	19.43	10.45	0.54	3.E-05	***
PE (18:1/20:4)	2.69	2.69	1.00	0.978	
PE (16:0/22:6)	7.35	8.00	1.09	0.374	
PE (16:1/22:6)	1.80	3.20	1.78	0.035	*
PE (18:0/22:6)	29.20	23.87	0.82	0.002	**
PE (18:1/22:6)	8.37	10.63	1.27	0.025	*
PE (18:2/22:6)	8.24	9.25	1.12	0.038	*

“Ratio” is the comparative value of the relative areas (old vs. young). ***p < 0.001, **p < 0.01, *p < 0.05.

A previous study had reported the fatty acid composition in soleus muscle (red, slow-twitch, oxidative fiber) and extensor digitorum longus muscle (EDL, white, fast-twitch, glycolytic fiber) in rat^13^. According to the study, soleus, compared with EDL, contained higher level of 18:0, 18:1(n-9), and 18:2(n-6) fatty acids and lower level of 16:0, and 22:6(n-3) fatty acids^13^. In our results, the level of phospholipid species composed of only 18:0, 18:1, and 18:2 were higher in aged mice than in young mice, while the level of phospholipid species composed of only16:0, and 22:6 were lower in aged mice than in young mice (Table 2). In particular, in the case of PC, the dominant phospholipid in skeletal muscle^7^, PC(18:1/18:1), PC(18:0/18:2), PC(18:1/18:2), and PC(18:2/18:2) levels were significantly higher and those of PC(16:0/16:0), and PC(16:0/22:6) significantly lower in aged than in young mice (Table 2). These findings are consistent with the observation that aged mice exhibited decreased glycolytic white fibers than young mice (Fig. 3c,e,f).

### Polyamines

**Figure 5:**
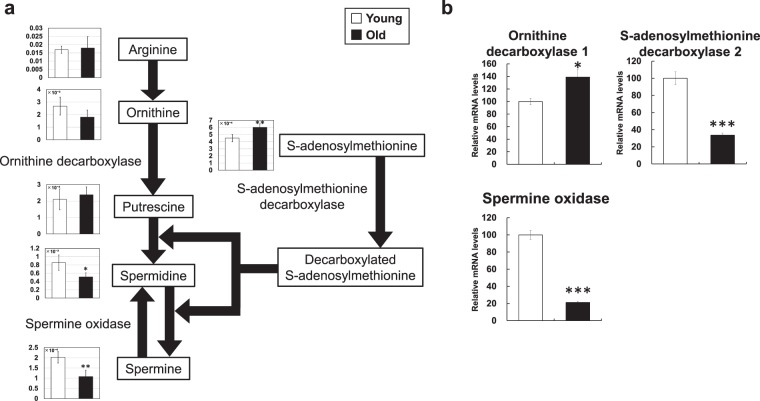
Metabolic changes related to polyamine metabolism. (**a**) Metabolite changes in the skeletal muscle of young and aged mice are shown. Relative metabolite changes shown in the graphs were obtained by CE-TOFMS (Supplementary Table 1). Open bars, young mice; filled bars, aged mice. Data are expressed as mean ± SD (N = 5); **p < 0.01, *p < 0.05. (**b**) Gene expression of polyamine metabolism in skeletal muscle from young and aged mice. Open bars, young mice; filled bars, aged mice. Data are expressed as mean ± SE (N = 5); ***p < 0.001, *p < 0.05.

Polyamines have various physiological functions, including roles in cell division, proliferation, and synthesis of nucleic acids and proteins^14,15^. As shown in Fig. 5a, putrescine, spermidine, and spermine are polyamines. Of these, spermidine (0.6-fold decrease) and spermine (0.5-fold decrease) were significantly altered in the skeletal muscle of aged mice (Fig. 5a). A previous study also reported decreased polyamine levels in various tissues of aged mice, including skeletal muscle^16^.

Ornithine is metabolized to putrescine by ornithine decarboxylase. The addition of propylamine to putrescine produces spermidine, and further propylamine addition produces spermine. Propylamine is supplied from S-adenosylmethionine catalyzed by S-adenosylmethionine decarboxylase. Spermine is metabolized to spermidine by spermine oxidase. In aged mice, the mRNA level of ornithine decarboxylase increased (1.4-fold), whereas those of S-adenosylmethionine decarboxylase and spermine oxidase decreased (0.3-fold and 0.2-fold, respectively) (Fig. 5b). Decreased S-adenosylmethionine decarboxylase and spermine oxidase expression can cause decreased spermidine and spermine levels, which may contribute to aged phenotypes such as decreased cell proliferation and nucleotide/protein synthesis. Indeed, knockdown of S-adenosylmethionine decarboxylase mRNA by siRNA in primary myoblast cells caused decreased cell proliferation assessed by EdU incorporation (Supplementary Fig. 2). Agents such as inhibitors of polyamine degradation enzymes or stimulators of polyamine synthesis enzymes could be candidates for drugs or functional foods to treat sarcopenia. This possibility remains to be pursued.

### Neurotransmitters

**Figure 6:**
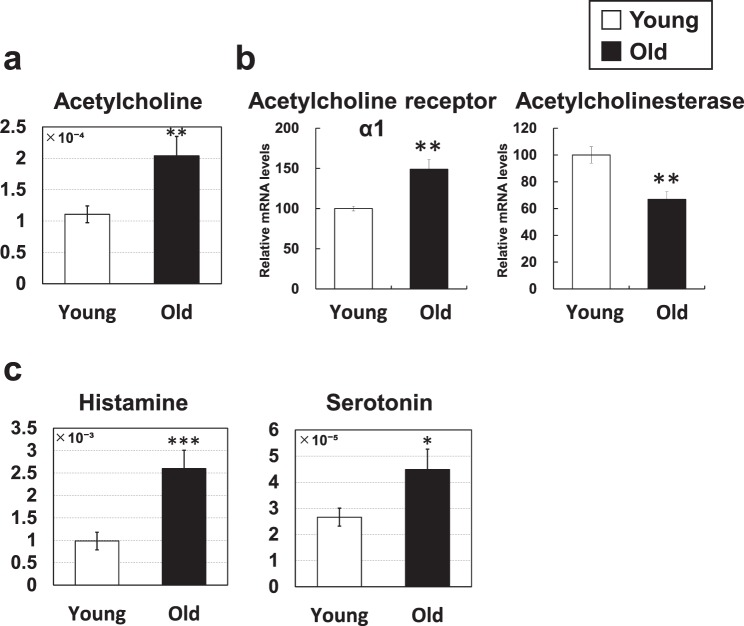
Metabolic changes related to neurotransmitters. (**a**,**c**) Metabolite changes in the skeletal muscle of young and aged mice are shown. Relative metabolite changes shown in the graphs were obtained by CE-TOFMS (Supplementary Table 1). Open bars, young mice; filled bars, aged mice. Data are expressed as mean ± SD (N = 5); ***p < 0.001, **p < 0.01, *p < 0.05. (**b**) Gene expression of acetylcholine receptor and acetylcholinesterase in skeletal muscle from young and aged mice. Open bars, young mice; filled bars, aged mice. Data are expressed as mean ± SE (N = 5); **p < 0.01.

Increased acetylcholine (1.8-fold) was observed in the skeletal muscle of aged mice (Fig. 6a). Acetylcholine is a neurotransmitter that is secreted from synaptic vesicles of motor nerve axon terminals in neuromuscular junctions during muscle contraction. During aging, neuromuscular junctions drop out and, as compensation, the expression levels of acetylcholine receptors increase, as determined by denervation experiments^17^. Moreover, neuromuscular junctions are known to degenerate during the aging process^9,10^. Thus, the increased acetylcholine level observed in this study (Fig. 6a) is likely the compensation for decreased neuromuscular junction signals. Indeed, we observed increased acetylcholine receptor mRNA expression (1.5-fold) in aged mice (Fig. 6b). Moreover, the mRNA level of acetylcholinesterase, a degradation enzyme of acetylcholine, was decreased in aged mice (0.7-fold) (Fig. 6b), which may contribute to the increase in acetylcholine levels.

In addition to acetylcholine, the neurotransmitters histamine (2.6-fold) and serotonin (1.7-fold) were increased in the skeletal muscle of aged mice (Fig. 6c). Physical damage to skeletal muscle can cause inflammation and induce the production of histamine, serotonin, and prostaglandins^18^. These substances affect nociceptive fibers and cause muscle pain^18^. In addition, muscle regeneration capacity decreases with age^8^. Together, age-induced muscle damage and delayed regeneration may increase histamine and serotonin levels.

### Others

**Figure 7:**
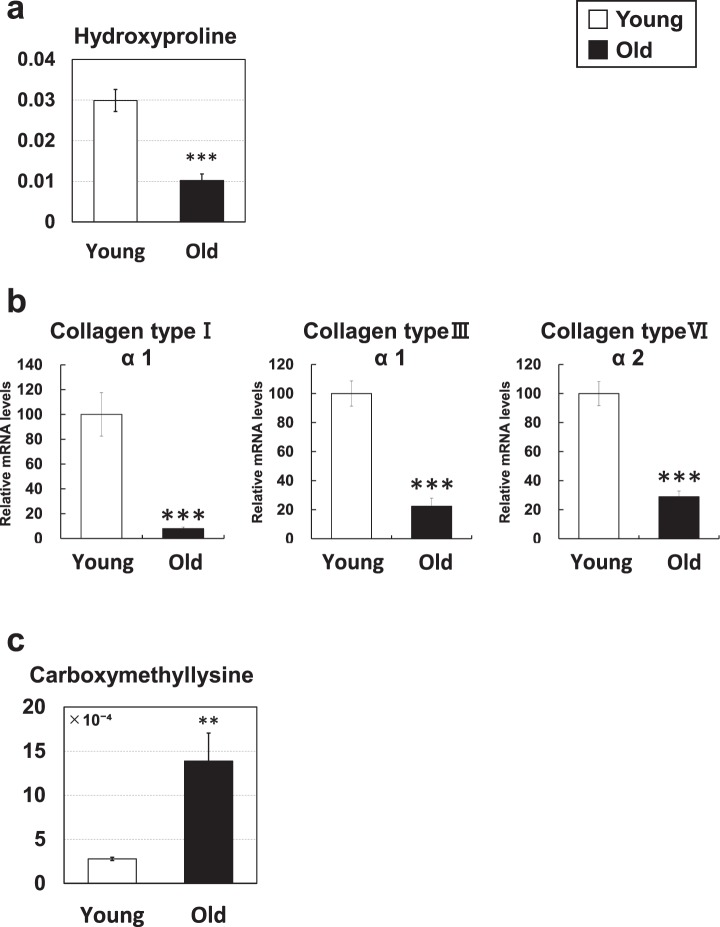
Metabolic changes related to amino acids. (**a**,**c**) Metabolite changes in the skeletal muscle of young and aged mice are shown. Relative metabolite changes shown in the graphs were obtained by CE-TOFMS (Supplementary Table 1). Open bars, young mice; filled bars, aged mice. Data are expressed as mean ± SD (N = 5); ***p < 0.001, **p < 0.01. (**b**) Gene expression of collagen in skeletal muscle from young and aged mice. Open bars, young mice; filled bars, aged mice. Data are expressed as mean ± SE (N = 5); ***p < 0.001.

Changes in several other amino acid-related metabolites were noted. The levels of hydroxyproline, a major component of collagen, were decreased in aged mice (0.3-fold) (Fig. 7a). The mRNA level of collagen type I α1 (0.1-fold), type III α1 (0.2-fold) and type VI α2 (0.3-fold), which are major collagens in skeletal muscle^19^, was markedly decreased in the skeletal muscle of aged mice (Fig. 7b). Thus, the decreased hydroxyproline levels may reflect the decreased collagen gene expression in aged mice.

Carboxylmethyllysine was markedly increased in aged mice (5.0-fold) (Fig. 7c). Carboxylmethyllysine is an advanced glycation end product (AGE) that is known to accumulate during the aging process^20^. In addition, the carboxylmethyllysine level is known to correlate with vascular diseases and arteriosclerosis in diabetic patients^21^. The increased carboxylmethyllysine is likely a phenotype of aged mice.

S-adenosylmethionine is a donor of methyl groups for DNA and protein. Global genomic DNA methylation levels increase in skeletal muscle in response to aging^22^. The increased S-adenosylmethionine level in aged muscle observed in this study (Fig. 5a) may be involved in the process of DNA methylation.

### Implications of sex difference in age-induced sarcopenia

In the current study, we used young and aged male mice for analysis. It has been reported that there are no differences in the incidence rate of sarcopenia in male and female humans^23^. On the contrary, other authors have reported that males are more susceptible to sarcopenia than are females^24^. Thus, there may be differences between the sexes with respect to sarcopenia susceptibility, but this has yet to be confirmed, and future studies are required to validate. Differences in metabolite profile in aged muscles between males and females (to evaluate if there was any sex difference between the young and old animals with regards to muscle metabolites) would be the next issue to be addressed.

## Conclusions

**Figure 8:**
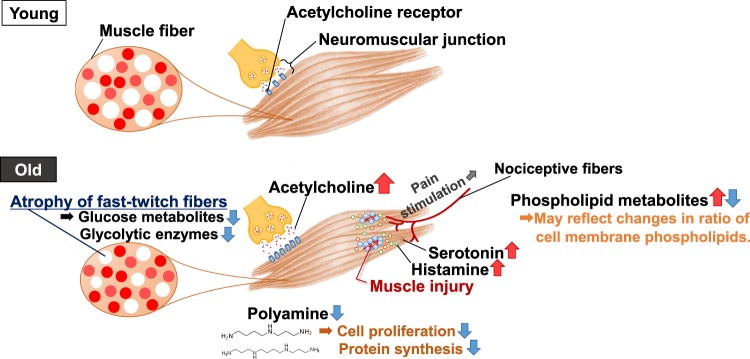
Schematic summary of this study. In the skeletal muscle of aged mice, several metabolite changes were observed. (1) Glucose metabolites decreased, likely due to preferential atrophy of fast-twitch fibers. (2) Phospholipid metabolites changed, likely reflecting changes in the ratio of cell membrane phospholipids. (3) Neurotransmitter levels significantly increased, likely because of neuromuscular junction dropout and muscle injury. (4) Products of polyamine metabolism decreased, probably contributing to aging phenotypes.

In this study, we observed various metabolite changes in aged mice (Fig. 8). The changed metabolites suggest the following: 1) decreases in the glycolytic pathway, possibly from decreased glycolytic muscle fibers; 2) changes in the phospholipid-related pathway that reflect muscle cellular membrane components during aging; and 3) aging-related neurodegeneration and muscle degeneration. Further analysis focusing on the altered metabolites observed in this study will provide essential data for understanding aging muscles.

## Methods

### Animals and ethics statement

C57BL/6J mice were purchased from Shimizu Laboratory Supplies (Kyoto, Japan). The mice were allowed ad libitum access to food (a standard laboratory diet, CE2, Clea, Tokyo, Japan) and water. All animal experiments were performed with the approval of the Institutional Animal Care and Use Committees of Kyoto Prefectural University. The protocol was approved by the Committee (No. KPU260407, review board: Dr. Yasuhiro Tsukamoto). All surgery was performed under sodium pentobarbital anesthesia, and all efforts were made to minimize suffering. The mice were cared for in accordance with the National Institutes of Health (NIH) Guide for the Care and Use of Laboratory Animals. All other experiments were performed in accordance with our institutional guidelines.

### Metabolomic analysis

Gastrocnemius muscles of 8-week-old male mice (N = 5, young group) and 28-month-old male mice (N = 5, aged group) were used for the metabolomic analysis (Human Metabolome Technologies Inc., Tsuruoka, Japan)^25–27^. Metabolomic analysis was performed as previously described^25^. The samples were subjected to CE-TOFMS analysis using the Agilent CE-TOFMS system (Agilent Technologies, Santa Clara, CA). The relative area of each peak was calculated and compared between the young and aged groups. The metabolomics data was submitted to Metabolomics Workbench (www.metabolomicsworkbench.org).

### Quantitative real-time RT-PCR analysis

Total RNA was isolated from tissue homogenates using the TRIzol reagent (Thermo Fisher Scientific Inc., Tokyo, Japan). cDNA was synthesized using 500 ng of each RNA sample with ReverTra Ace qPCR RT Master Mix Transcription Kit (Toyobo, Tokyo, Japan). Gene expression levels was measured as described previously^8^. The mouse-specific primer pairs used were as shown in Supplementary Table 3.

### Immunohistochemical analysis

Immunohistochemical analysis was performed on tibilias anterior muscle of young (4-month-old, N = 6) and aged (27-month-old, N = 7) mice as previously described^28^. Primary antibodies used were as follows: mouse anti-type IIa myosin heavy chain (MyHC) (SC-71), mouse anti-type IIb MyHC (BF-F3), mouse anti-type I MyHC (BA-D5) antibodies were obtained from Deutsche Sammlung von Mikroorganismen (Braunschweig, Germany). Rat anti-Laminin α2 antibody was obtained from Enzo (Enzo Life Sciences, NY, USA). Immuno-stained images were optimized globally and assembled into figures with Photoshop (Adobe, San Diego, CA, USA). Minimum fiber Feret’s diameter measurement^12^ was performed using ImageJ/Fiji software (https://imagej.nih.gov/ij/docs/guide/index.html). Samples with significant staining artifacts were excluded from automated analyses.

### Liquid chromatography-mass spectrometry (LC-MS)

Lipids were extracted from the extensor digitorum longus muscle (EDL) of young and aged mice. The mice were same as used in CE-TOFMS. LC-MS analysis was performed as described before^7^, with slight modification. Namely, phospholipid fractions were analyzed using an LCMS-8040 triple–quadrupole mass spectrometer (Shimadzu, Kyoto, Japan) equipped with an electrospray source ionization probe. For high-performance liquid chromatography (HPLC) analysis, an Accucore RP-MS C18 column (2.6 μm, 2.1 × 50 mm, Thermo Fisher Scientific) was used. To quantify phosphatidylcholine (PC) and phosphatidylethanolamine (PE), multiple reaction monitoring was performed with the transitions [M + HCOO]− to [RCOO]− for PC, [M − H]− to [RCOO]− for PE. Peak areas of each individual species were normalized against the sum of all peak areas within each phospholipid class to determine the relative abundances (expressed as % of total). After applying autoscaling, mean-centering, and scaling by standard deviation on a per-peak basis as pretreatment, a principal component analysis (PCA) was conducted using JMP ver. 11 (SAS Institute, Cary, NC, USA).

### Primary myoblast cell culture

Primary myoblast cell isolation and culture was performed as described before^8^. Measuring cell proliferation, we used the Click-iT Plus 5-ethynyl-2′-deoxyuridine (EdU) Alexa Fluor 488 Flow Cytometry Assay Kit (Thermo Fisher Scientific)^8^. siRNA of S-adenosylmethionine decarboxylase mRNA was purchased from Sigma-Aldrich Japan; MISSION siAmd2 (SASI_Mm02_00311615), and MISSION siRNA Universal Negative Control #1. Lipofectamine RNAiMax (Thermo Fisher Scientific) was used for transfection of siRNA.

### cDNA microarray analysis

Gastrocnemius muscles of 13-week-old male mice (N = 3, young group) and 26-month-old male mice (N = 3, aged group) were used for the microarray analysis. The average body weight was 23.5 ± 1.7 g for young mice and 29.5 ± 2.5 g for aged mice. The average muscle (gastrocnemius) weight was 139.1 ± 7.6 mg for young and 122.8 ± 11.8 mg for aged mice. cDNA microarray data were collected as described previously^25^. The microarray data was submitted to GEO database (accession No. GSE125815).

## Supplementary information

Supplementary information

Author signitures
