# Supplementary material for: Metabolomic Analysis of Skeletal Muscle in Aged Mice
Source: Sci Rep. 2019 Jul 18;9:10425. doi: 10.1038/s41598-019-46929-8 (PMC6639307; doi:10.1038/s41598-019-46929-8)
Supplement: Supplementary file 1 — Supplementary information [file 41598_2019_46929_MOESM1_ESM.pdf]

Metabolomic Analysis of Skeletal Muscle in Aged Mice

Ran Uchitomi[1], Yukino Hatazawa[1], Nanami Senoo[2], Kiyoshi Yoshioka[3], Mariko Fujita[1], Takahiko Shimizu[4], Shinji Miura[2], Yusuke Ono[3], Yasutomi Kamei[1]*

[1] Graduate School of Life and Environmental Sciences, Kyoto Prefectural University, Kyoto, Japan.
[2] Laboratories of Nutritional Biochemistry, Graduate School of Nutritional and Environmental Sciences, University of Shizuoka, Shizuoka, Japan.
[3] Institute of Molecular Embryology and Genetics, Kumamoto University, Kumamoto, Japan.
[4] Department of Advanced Aging Medicine, Chiba University Graduate School of Medicine, Chiba, Japan.

*Corresponding author: Yasutomi Kamei, Professor, Laboratory of Molecular Nutrition, Graduate School of Life and Environmental Sciences, Kyoto Prefectural University, Kyoto 606-8522 Japan, E-mail: kamei@kpu.ac.jp

Supplementary Table 1. List of metabolites detected in CE-TOFMS.

"Relative area" is the peak value of each metabolite normalized by the sample volume, i.e., the relative concentration of each metabolite. "Mean" is the mean value of the relative area from each group (young and old [N = 5]). "Ratio" is the comparative value of the relative areas (old vs. young). "Not detected in young" or "Not detected in old" indicates that the peak of the metabolite was below the detection level in that group. The p-value was calculated using Welch's t-test (***p < 0.001, **p < 0.01, *p < 0.05).

Compound name	Relative Area												Comparative Analysis		
	Young					Old					Mean		Old vs Young		
	Young-1	Young-2	Young-3	Young-4	Young-5	Old-1	Old-2	Old-3	Old-4	Old-5	Young	Old	Ratio	*p* -value	
2-Hydroxyisobutyric acid	-	-	-	-	-	-	-	-	-	0.00014	-	0.00014	not detected in Young	-	
2-Oxoisovaleric acid	-	-	-	-	-	-	0.00030	-	-	-	-	0.00030	not detected in Young	-	
Cholic acid	-	-	-	-	-	-	-	-	0.00129	-	-	0.00129	not detected in Young	-	
N-Acetyllysine	-	-	-	-	-	-	0.00026	0.00025	0.00035	-	-	0.00029	not detected in Young	-	
Octanoic acid	-	-	-	-	-	0.00019	-	-	0.00021	-	-	0.00020	not detected in Young	-	
S-Methylmethionine	-	-	-	-	-	0.00006	-	-	-	-	-	0.00006	not detected in Young	-	
Taurocholic acid	-	-	-	-	-	-	-	-	0.00035	-	-	0.00035	not detected in Young	-	
Carboxymethyllysine	0.00030	0.00026	0.00027	0.00029	0.00026	0.00185	0.00155	0.00133	0.00109	0.00111	0.00028	0.00139	5.00	0.0015	**
1-Methylhistamine	0.00004	0.00003	0.00003	0.00003	0.00003	0.00019	0.00020	0.00012	0.00017	0.00015	0.00003	0.00017	4.95	0.0008	***
Histamine	0.00096	0.00130	0.00091	0.00099	0.00076	0.00251	0.00285	0.00223	0.00317	0.00223	0.00098	0.00260	2.64	0.0003	***
Diethanolamine	0.00016	0.00022	0.00031	0.00017	0.00016	0.00070	0.00075	0.00038	0.00036	0.00030	0.00020	0.00050	2.45	0.0312	*
Pantothenic acid	0.00040	0.00034	0.00034	0.00040	0.00041	0.00069	0.00059	0.00070	0.00099	0.00062	0.00038	0.00072	1.90	0.0079	**
Thiamine	-	-	0.00002	0.00002	-	0.00004	0.00004	-	-	0.00004	0.00002	0.00004	1.86	0.0021	**
Homocarnosine	0.04865	0.04346	0.05429	0.03809	0.04439	0.07392	0.08117	0.08164	0.08955	0.09777	0.04578	0.08481	1.85	0.0001	***
Acetylcholine	0.00011	0.00012	0.00012	0.00009	-	0.00019	0.00017	0.00021	0.00020	0.00025	0.00011	0.00020	1.84	0.0011	**
Guanosine	0.00003	0.00006	-	0.00004	0.00003	0.00006	0.00007	0.00006	0.00011	0.00005	0.00004	0.00007	1.79	0.0340	*
Argininosuccinic acid	0.00018	0.00019	0.00016	0.00015	0.00013	0.00030	0.00044	0.00012	0.00031	0.00026	0.00016	0.00029	1.77	0.0735	
N8-Acetylspermidine	-	0.00003	0.00003	-	-	0.00004	-	-	-	0.00005	0.00003	0.00005	1.71	0.0698	
Serotonin	0.00002	-	0.00003	0.00003	-	-	0.00006	0.00004	0.00004	0.00004	0.00003	0.00004	1.69	0.0119	*
Cysteine glutathione disulfide	0.00003	0.00003	0.00004	0.00002	0.00003	0.00005	0.00004	0.00005	0.00007	0.00003	0.00003	0.00005	1.65	0.0185	*
myo-Inositol 1-phosphate<br>myo-Inositol 3-phosphate	0.00145	0.00134	0.00132	0.00104	0.00109	0.00184	0.00189	0.00213	0.00228	0.00199	0.00125	0.00203	1.63	0.0001	***
IMP	0.00728	0.00452	0.00315	0.00541	0.00312	0.01219	0.00489	0.00512	0.00517	0.01018	0.00469	0.00751	1.60	0.1534	
Nω-Methylarginine	0.00006	-	-	-	-	-	0.00009	-	-	-	0.00006	0.00009	1.53	-	
Urocanic acid	0.00033	0.00009	-	0.00011	0.00013	-	0.00027	0.00018	0.00029	-	0.00017	0.00025	1.48	0.2633	
Inosine	0.00111	0.00138	0.00070	0.00103	0.00077	0.00185	0.00149	0.00119	0.00129	0.00152	0.00100	0.00147	1.47	0.0224	*
XC0137	0.00011	0.00011	0.00015	0.00010	0.00007	0.00016	0.00019	0.00011	0.00014	0.00019	0.00011	0.00016	1.44	0.0434	*
4-Methyl-2-oxovaleric acid<br>3-Methyl-2-oxovaleric acid	0.00043	-	-	0.00029	-	0.00062	0.00055	-	0.00040	0.00047	0.00036	0.00051	1.41	0.2381	
Glycerophosphocholine	0.00267	0.00458	0.00328	0.00368	0.00307	0.00388	0.00472	0.00538	0.00506	0.00494	0.00346	0.00480	1.39	0.0124	*
Stachydrine	0.00249	0.00189	0.00178	0.00172	0.00140	0.00226	0.00295	0.00232	0.00227	0.00275	0.00185	0.00251	1.36	0.0211	*
Adenosine	0.00090	0.00168	0.00089	0.00117	0.00095	0.00161	0.00182	0.00117	0.00148	0.00137	0.00112	0.00149	1.34	0.0807	
S-Adenosylmethionine	0.00051	0.00046	0.00045	0.00047	0.00037	0.00063	0.00059	0.00063	0.00064	0.00052	0.00045	0.00060	1.33	0.0015	**
Uric acid	0.00034	0.00024	0.00024	0.00022	0.00023	0.00039	0.00029	0.00025	0.00037	0.00041	0.00026	0.00034	1.33	0.0547	
Citric acid	0.00826	0.00891	0.00662	0.00733	0.00721	0.01005	0.01001	0.00943	0.00936	0.01205	0.00767	0.01018	1.33	0.0045	**
XC0016	0.00028	0.00021	0.00025	0.00026	0.00027	0.00033	0.00025	0.00032	0.00037	0.00039	0.00025	0.00033	1.32	0.0265	*
Phe	0.01129	0.01290	0.01128	0.01005	0.01120	0.01277	0.01562	0.01110	0.01742	0.01724	0.01134	0.01483	1.31	0.0468	*
p-Toluic acid<br>m-Toluic acid<br>o-Toluic acid	0.00034	0.00019	0.00016	0.00023	-	0.00035	0.00028	0.00025	0.00032	0.00029	0.00023	0.00030	1.30	0.1691	
XA0017	0.00015	-	0.00011	-	-	-	-	-	0.00017	-	0.00013	0.00017	1.30	-	
Glu-Glu	0.00004	-	-	0.00004	-	0.00004	0.00005	0.00004	0.00007	-	0.00004	0.00005	1.29	0.1901	
Cytidine	0.00014	0.00017	0.00017	0.00017	0.00012	0.00016	0.00014	0.00026	0.00027	0.00016	0.00015	0.00020	1.28	0.2118	

N-Acetylaspartic acid	0.00033	0.00072	0.00037	0.00048	0.00037	0.00045	0.00040	0.00066	0.00074	0.00064	0.00045	0.00058	1.27	0.2356	
2-Hydroxyglutaric acid	0.00074	0.00064	0.00052	0.00055	0.00052	0.00076	0.00076	0.00058	0.00063	0.00098	0.00059	0.00074	1.26	0.1098	
His	0.00956	0.01006	0.01162	0.01097	0.00981	0.01181	0.01205	0.01121	0.01726	0.01285	0.01040	0.01304	1.25	0.0717	
Threonic acid	0.00018	0.00016	0.00016	0.00020	0.00020	0.00026	0.00025	0.00024	0.00018	0.00021	0.00018	0.00023	1.25	0.0329	*
Isethionic acid	0.00042	0.00035	0.00033	0.00035	0.00037	0.00047	0.00047	0.00041	0.00044	0.00048	0.00036	0.00046	1.25	0.0017	**
XA0065	0.00018	0.00020	0.00016	0.00020	0.00018	0.00022	0.00019	0.00028	0.00025	0.00019	0.00018	0.00023	1.25	0.0631	
Tyr	0.00886	0.01087	0.00900	0.00701	0.01191	0.01201	0.01478	0.00784	0.01183	0.01262	0.00953	0.01181	1.24	0.1468	
Trp	0.00219	0.00228	0.00207	0.00170	0.00185	0.00233	0.00268	0.00191	0.00295	0.00261	0.00202	0.00250	1.24	0.0558	
Gln	0.10350	0.11148	0.12828	0.10624	0.11397	0.13057	0.13534	0.13681	0.16310	0.12758	0.11269	0.13868	1.23	0.0114	*
Ethanolamine phosphate	0.00070	0.00098	0.00073	0.00080	0.00066	0.00097	0.00092	0.00100	0.00105	0.00079	0.00077	0.00095	1.22	0.0434	*
Fructose 6-phosphate	0.02243	0.00846	0.01966	0.00784	0.01139	0.01812	0.01488	0.01479	0.01224	0.02514	0.01396	0.01703	1.22	0.4351	
Glucose 6-phosphate	0.08798	0.03265	0.06730	0.03138	0.04449	0.06923	0.05441	0.05237	0.04520	0.09792	0.05276	0.06382	1.21	0.4645	
GABA	0.00043	-	-	-	-	0.00053	-	-	0.00047	0.00056	0.00043	0.00052	1.21	-	
3-Hydroxybutyric acid	0.00146	0.00144	0.00118	0.00096	0.00112	0.00218	0.00127	0.00104	0.00113	0.00181	0.00123	0.00149	1.21	0.3331	
Trimethylamine N-oxide	0.00056	0.00049	0.00053	0.00056	0.00055	0.00072	0.00071	0.00060	0.00031	0.00086	0.00054	0.00064	1.19	0.3347	
CMP-N-acetylneuraminate	0.00021	0.00018	0.00020	0.00017	0.00023	0.00025	0.00022	0.00025	0.00023	0.00022	0.00020	0.00023	1.19	0.0214	*
Urea	0.28760	0.26410	0.23429	0.22951	0.21853	0.29611	0.33959	0.25912	0.16226	0.38700	0.24681	0.28881	1.17	0.3455	
1-Aminocyclopropane-1-carboxylic acid<br>Homoserinelactone	0.00011	0.00013	0.00010	0.00009	-	0.00013	0.00013	0.00008	0.00015	0.00013	0.00011	0.00012	1.16	0.2178	
UDP-glucose<br>UDP-galactose	0.00060	0.00051	0.00063	0.00061	0.00057	0.00059	0.00061	0.00073	0.00070	0.00077	0.00059	0.00068	1.16	0.0556	
5'-Deoxy-5'-methylthioadenosine	0.00004	0.00004	0.00005	0.00003	0.00002	0.00005	0.00004	0.00003	0.00005	0.00003	0.00004	0.00004	1.16	0.3474	
Hypoxanthine	0.00060	0.00066	0.00037	0.00054	0.00041	0.00074	0.00065	0.00050	0.00054	0.00055	0.00052	0.00060	1.16	0.2722	
Lauric acid	0.00048	0.00048	0.00049	0.00049	0.00043	0.00059	0.00067	0.00054	0.00052	0.00042	0.00047	0.00055	1.16	0.1494	
Glucose 1-phosphate	0.00514	0.00208	0.00436	0.00193	0.00269	0.00408	0.00313	0.00301	0.00273	0.00572	0.00324	0.00374	1.15	0.5722	
Glutathione (GSSG)_divalent	0.02384	0.01825	0.02639	0.02555	0.02500	0.03077	0.03096	0.02759	0.02303	0.02481	0.02381	0.02743	1.15	0.1296	
5-Oxoproline	0.00031	0.00019	0.00018	0.00021	0.00021	0.00022	0.00031	0.00023	0.00034	0.00017	0.00022	0.00025	1.15	0.4270	
N,N-Dimethylglycine	0.00071	-	-	-	-	0.00081	-	-	-	-	0.00071	0.00081	1.14	-	
Gly-Gly	0.00021	0.00017	0.00020	0.00016	0.00026	0.00018	0.00015	0.00027	0.00028	0.00026	0.00020	0.00023	1.14	0.4084	
Hypotaurine	0.00416	0.00510	0.00443	0.00363	0.00289	0.00353	0.00398	0.00358	0.00931	0.00258	0.00404	0.00459	1.14	0.6782	
Leu	0.02022	0.03128	0.02519	0.02382	0.02119	0.02892	0.02913	0.02309	0.02564	0.03157	0.02434	0.02767	1.14	0.2135	
Putrescine	0.00013	0.00017	0.00020	0.00026	0.00028	0.00023	0.00018	0.00028	0.00021	0.00029	0.00021	0.00024	1.13	0.4642	
Ile	0.01297	0.01831	0.01438	0.01338	0.01142	0.01764	0.01556	0.01508	0.01488	0.01625	0.01409	0.01588	1.13	0.2110	
UDP-N-acetylgalactosamine<br>UDP-N-acetylglucosamine	0.00057	0.00059	0.00056	0.00063	0.00051	0.00062	0.00050	0.00067	0.00087	0.00055	0.00057	0.00064	1.12	0.3460	
Pelargonic acid	0.00037	0.00028	0.00026	0.00025	0.00034	0.00034	0.00032	0.00029	0.00039	0.00036	0.00030	0.00034	1.12	0.2375	
Triethanolamine	0.00006	-	0.00010	-	0.00011	0.00007	-	0.00010	-	0.00014	0.00009	0.00010	1.12	0.6834	
FAD_divalent	-	0.00019	0.00015	-	0.00018	0.00018	0.00020	0.00017	0.00021	0.00019	0.00017	0.00019	1.11	0.1972	
Fumaric acid	0.00215	0.00206	0.00164	0.00167	0.00170	0.00247	0.00230	0.00187	0.00158	0.00201	0.00184	0.00205	1.11	0.3223	
Betaine	0.01011	0.01315	0.01104	0.00881	0.00826	0.00965	0.01115	0.01072	0.01694	0.00838	0.01027	0.01137	1.11	0.5433	
Methionine sulfoxide	0.00049	0.00042	0.00044	0.00049	0.00034	0.00070	0.00047	0.00025	0.00027	0.00069	0.00043	0.00048	1.10	0.6920	
Malic acid	0.01773	0.01576	0.01148	0.01412	0.01317	0.01891	0.01770	0.01481	0.01216	0.01571	0.01445	0.01586	1.10	0.4016	
5-Hydroxylysine	0.00013	0.00018	0.00016	0.00020	0.00013	0.00018	0.00023	-	0.00010	0.00019	0.00016	0.00018	1.09	0.6583	
Taurine	0.24377	0.26238	0.25518	0.24311	0.24839	0.26910	0.27878	0.24779	0.27494	0.28651	0.25057	0.27142	1.08	0.0305	*
Gly-Asp	0.00021	0.00027	0.00028	0.00025	0.00023	0.00030	0.00024	0.00031	0.00027	0.00025	0.00025	0.00027	1.08	0.3114	
γ-Butyrobetaine	0.00304	0.00266	0.00271	0.00267	0.00280	0.00240	0.00307	0.00263	0.00402	0.00277	0.00278	0.00298	1.07	0.5181	
Malonylcarnitine	0.00007	0.00007	0.00006	0.00009	0.00007	-	0.00011	0.00006	0.00006	0.00008	0.00007	0.00008	1.07	0.6705	
NADP+	0.00027	0.00030	0.00029	0.00026	0.00023	0.00030	0.00029	0.00024	0.00033	0.00027	0.00027	0.00029	1.07	0.3571	
Terephthalic acid	0.00029	0.00033	0.00028	0.00029	0.00032	0.00033	0.00033	0.00034	0.00030	0.00030	0.00030	0.00032	1.07	0.1500	
Arg	0.01476	0.01860	0.01943	0.01710	0.01470	0.01866	0.02445	0.00697	0.02316	0.01660	0.01692	0.01797	1.06	0.7603	
Succinic acid	0.01206	0.00988	0.01010	0.01045	0.01203	0.01314	0.00949	0.01080	0.01170	0.01233	0.01090	0.01149	1.05	0.4772	

Pyridoxamine 5'-phosphate	0.00019	0.00025	0.00019	0.00024	0.00024	0.00022	0.00029	0.00022	0.00025	0.00020	0.00022	0.00023	1.05	0.5774	
XA0027	0.00019	-	-	0.00018	-	0.00018	-	0.00016	0.00024	-	0.00019	0.00019	1.04	0.7913	
Pipecolic acid	0.00140	0.00137	0.00137	0.00148	0.00133	0.00137	0.00193	0.00128	0.00086	0.00175	0.00139	0.00144	1.03	0.8151	
4-Guanidinobutyric acid	0.00012	-	-	0.00012	0.00012	-	0.00013	0.00012	-	0.00013	0.00012	0.00013	1.03	0.3233	
Trigonelline	0.00047	0.00043	0.00045	0.00052	0.00032	0.00040	0.00056	0.00034	0.00029	0.00065	0.00044	0.00045	1.03	0.8848	
trans-Glutaconic acid	0.00068	-	-	-	-	0.00064	0.00074	-	0.00052	0.00089	0.00068	0.00070	1.03	-	
Glycerol	0.08445	0.06145	0.08006	0.05397	0.05179	0.04782	0.08066	0.07566	0.05811	0.07765	0.06634	0.06798	1.02	0.8644	
S-Adenosylhomocysteine	0.00005	0.00004	0.00006	0.00004	0.00005	0.00006	0.00006	0.00004	0.00004	0.00004	0.00005	0.00005	1.02	0.8863	
Carnosine	0.08153	0.08073	0.11021	0.07015	0.08910	0.08881	0.09044	0.08470	0.07044	0.10625	0.08635	0.08813	1.02	0.8448	
2'-Deoxycytidine	0.00007	0.00006	0.00006	0.00005	0.00005	0.00005	0.00005	0.00006	0.00006	0.00007	0.00006	0.00006	1.02	0.8681	
Val	0.02642	0.03816	0.03349	0.02969	0.02984	0.03411	0.03663	0.02649	0.02776	0.03504	0.03152	0.03200	1.02	0.8702	
Ser	0.01479	0.01779	0.02292	0.01926	0.01871	0.01699	0.01935	0.01717	0.02384	0.01718	0.01869	0.01891	1.01	0.9115	
CoA_divalent	0.00040	0.00050	0.00044	0.00033	0.00030	0.00037	0.00038	0.00036	0.00045	0.00042	0.00039	0.00040	1.01	0.9173	
Lactic acid	0.22559	0.21733	0.16937	0.23153	0.22475	0.24335	0.22833	0.18630	0.19570	0.21935	0.21371	0.21461	1.00	0.9552	
N6,N6,N6-Trimethyllysine	0.00094	0.00111	0.00128	0.00133	0.00118	0.00105	0.00139	0.00118	0.00122	0.00102	0.00117	0.00117	1.00	0.9722	
CTP	0.00093	0.00094	0.00093	0.00091	0.00102	0.00100	0.00086	0.00103	0.00090	0.00095	0.00095	0.00095	1.00	0.9883	
Asp	0.00585	0.00782	0.00780	0.00770	0.00558	0.00543	0.01024	0.00779	0.00520	0.00597	0.00695	0.00692	1.00	0.9813	
1-Methylhistidine<br>3-Methylhistidine	0.00153	0.00151	0.00175	0.00167	0.00135	0.00137	0.00184	0.00145	0.00125	0.00184	0.00156	0.00155	0.99	0.9455	
XC0071	-	0.00004	-	0.00006	-	-	-	-	0.00005	-	0.00005	0.00005	0.99	-	
SDMA	-	0.00007	0.00008	0.00008	0.00009	-	-	0.00008	0.00008	-	0.00008	0.00008	0.99	0.8327	
Glu	0.05847	0.07433	0.06179	0.06015	0.05571	0.05755	0.08559	0.06167	0.04869	0.05287	0.06209	0.06127	0.99	0.9135	
S-Methylcysteine	0.00013	0.00013	0.00015	0.00012	0.00014	0.00012	0.00015	0.00017	0.00009	-	0.00013	0.00013	0.99	0.9217	
Adenine	0.00042	0.00039	0.00039	0.00033	0.00035	0.00045	0.00031	0.00032	0.00041	0.00035	0.00038	0.00037	0.98	0.8230	
Asn	0.00351	0.00432	0.00552	0.00460	0.00504	0.00379	0.00435	0.00370	0.00619	0.00440	0.00460	0.00448	0.97	0.8433	
Citrulline	0.00931	0.01003	0.00935	0.00773	0.00749	0.00737	0.01014	0.00653	0.01134	0.00739	0.00878	0.00855	0.97	0.8346	
Uridine	0.00028	0.00024	-	0.00023	0.00021	0.00023	0.00021	0.00022	0.00026	-	0.00024	0.00023	0.97	0.6424	
1-Methyladenosine	0.00004	0.00004	0.00004	0.00004	0.00005	0.00004	-	0.00004	0.00005	-	0.00004	0.00004	0.97	0.6790	
Homocitrulline	0.00044	0.00046	0.00042	0.00044	0.00039	0.00044	0.00048	0.00046	0.00034	0.00036	0.00043	0.00041	0.96	0.6041	
Ethanolamine	0.00127	0.00120	0.00145	0.00132	0.00137	0.00080	0.00099	0.00116	0.00249	0.00090	0.00132	0.00127	0.96	0.8757	
ADP	0.00217	0.00239	0.00172	0.00258	0.00214	0.00212	0.00181	0.00271	0.00216	0.00174	0.00220	0.00211	0.96	0.7026	
Met	0.00463	0.00647	0.00710	0.00479	0.00575	0.00618	0.00615	0.00409	0.00515	0.00597	0.00575	0.00551	0.96	0.7110	
Creatinine	0.00409	0.00378	0.00434	0.00382	0.00376	0.00324	0.00364	0.00376	0.00351	0.00473	0.00396	0.00378	0.95	0.5394	
Creatine	0.07496	0.07373	0.06539	0.07720	0.07329	0.07184	0.07069	0.06506	0.06902	0.07088	0.07291	0.06950	0.95	0.1893	
Choline	0.00953	0.01143	0.01097	0.00903	0.00939	0.00805	0.00688	0.01023	0.01684	0.00587	0.01007	0.00957	0.95	0.8170	
4-Methylpyrazole	-	-	0.00016	-	0.00011	-	0.00011	-	0.00013	0.00014	0.00013	0.00013	0.95	0.8350	
ADMA	0.00018	0.00016	0.00018	0.00015	0.00015	0.00017	0.00019	0.00013	0.00016	0.00013	0.00017	0.00016	0.95	0.5434	
O-Acetylhomoserine<br>2-Aminoadipic acid	0.00050	0.00039	0.00049	0.00044	0.00038	0.00038	0.00047	0.00046	0.00029	0.00048	0.00044	0.00042	0.95	0.6193	
O-Acetylcarnitine	0.03154	0.03172	0.02825	0.02839	0.02831	0.02612	0.03010	0.02491	0.02871	0.02990	0.02964	0.02795	0.94	0.2373	
Diethylaminomalonic acid	0.00028	0.00028	0.00033	0.00031	0.00033	0.00023	0.00034	0.00027	0.00030	0.00031	0.00031	0.00029	0.94	0.4088	
Dyphylline	0.00082	0.00055	0.00058	0.00059	0.00045	0.00049	0.00053	0.00052	0.00048	0.00078	0.00060	0.00056	0.94	0.6542	
Thr	0.01681	0.02521	0.02783	0.02326	0.02071	0.02365	0.02388	0.01793	0.01828	0.02265	0.02277	0.02128	0.93	0.5384	
ATP	0.21578	0.20119	0.21291	0.20313	0.20465	0.18814	0.19016	0.19749	0.19533	0.18738	0.20753	0.19170	0.92	0.0026	**
GTP	0.00294	0.00305	0.00325	0.00279	0.00274	0.00259	0.00245	0.00313	0.00291	0.00252	0.00295	0.00272	0.92	0.1775	
Nicotinamide	0.01081	0.01288	0.01033	0.01399	0.01329	0.01118	0.01316	0.01027	0.01021	0.01128	0.01226	0.01122	0.91	0.2794	
Ribulose 5-phosphate	0.00053	0.00058	0.00037	0.00051	0.00043	0.00056	0.00045	0.00038	0.00037	0.00043	0.00048	0.00044	0.90	0.3750	
Phosphocreatine	0.08845	0.08149	0.12987	0.07547	0.08883	0.06600	0.09141	0.08388	0.08983	0.08680	0.09282	0.08358	0.90	0.4194	
Carnitine	0.02655	0.02967	0.02794	0.02903	0.02585	0.01822	0.02865	0.02189	0.02806	0.02656	0.02781	0.02467	0.89	0.2007	
Homoserine	0.00020	0.00036	0.00030	0.00027	-	-	0.00025	-	-	-	0.00028	0.00025	0.89	-	
2-Hydroxyvaleric acid	0.00023	0.00022	0.00022	0.00019	0.00018	0.00021	0.00017	0.00017	0.00017	0.00017	0.00021	0.00018	0.87	0.0742	

Pro	0.02409	0.03367	0.03548	0.02642	0.02866	0.02403	0.02865	0.02400	0.02684	0.02521	0.02966	0.02574	0.87	0.1493	
N5-Ethylglutamine	0.00070	0.00078	0.00085	0.00061	0.00078	0.00068	0.00075	0.00056	0.00059	0.00054	0.00074	0.00062	0.84	0.0657	
Ala	0.10116	0.12221	0.14222	0.12591	0.12599	0.09392	0.11248	0.10796	0.09468	0.10463	0.12350	0.10273	0.83	0.0314	*
Glutathione (GSH)	0.02466	0.02757	0.02290	0.02064	0.01490	0.01737	0.01788	0.01973	0.01543	0.02043	0.02213	0.01817	0.82	0.1430	
Ribose 5-phosphate	0.00023	-	-	0.00020	0.00028	0.00019	-	-	-	-	0.00024	0.00019	0.82	-	
Glycerol 3-phosphate	0.02541	0.02175	0.02292	0.02174	0.02180	0.01678	0.02120	0.01754	0.01594	0.02022	0.02272	0.01833	0.81	0.0091	**
PRPP	0.00034	0.00045	0.00037	0.00032	0.00049	0.00041	0.00035	0.00032	0.00021	0.00026	0.00040	0.00031	0.79	0.1098	
Butyrylcarnitine	0.00010	0.00019	0.00033	-	0.00023	-	0.00011	-	0.00023	0.00016	0.00022	0.00017	0.79	0.4712	
Lys	0.03336	0.04598	0.04817	0.04285	0.03533	0.03849	0.03803	0.01889	0.03498	0.03031	0.04114	0.03214	0.78	0.0905	
UTP	0.00439	0.00421	0.00435	0.00431	0.00449	0.00310	0.00304	0.00382	0.00356	0.00343	0.00435	0.00339	0.78	0.0017	**
2-Aminoisobutyric acid<br>2-Aminobutyric acid	0.00104	0.00146	0.00138	0.00075	0.00104	0.00078	0.00089	0.00080	0.00072	0.00123	0.00113	0.00088	0.78	0.1556	
ADP-ribose	0.00178	0.00198	0.00146	0.00227	0.00265	0.00165	0.00182	0.00142	0.00131	0.00162	0.00203	0.00157	0.77	0.0882	
NAD+	0.01153	0.01075	0.01180	0.01179	0.01107	0.00847	0.00835	0.00868	0.00921	0.00891	0.01139	0.00872	0.77	0.0000	***
XC0061	0.00055	0.00059	0.00045	0.00055	0.00045	0.00036	0.00050	0.00031	0.00039	0.00040	0.00052	0.00039	0.75	0.0181	*
XA0033	0.00025	0.00034	0.00034	0.00035	0.00024	-	0.00022	-	0.00024	0.00023	0.00031	0.00023	0.75	0.0308	*
XC0132	0.00025	0.00023	0.00020	0.00021	0.00020	0.00014	0.00018	0.00014	0.00018	0.00017	0.00022	0.00016	0.75	0.0045	**
S-Lactoylglutathione	0.00038	0.00046	0.00020	0.00036	0.00020	0.00020	0.00020	0.00036	0.00020	0.00022	0.00032	0.00024	0.74	0.2161	
Gly	0.12393	0.11469	0.14076	0.10845	0.11607	0.08372	0.10211	0.10205	0.08693	0.06239	0.12078	0.08744	0.72	0.0076	**
Sarcosine	0.00142	0.00122	0.00141	0.00152	0.00133	0.00109	0.00092	0.00100	0.00065	0.00103	0.00138	0.00094	0.68	0.0020	**
Ornithine	0.00197	0.00341	0.00346	0.00233	0.00221	0.00194	0.00221	0.00115	0.00127	0.00242	0.00268	0.00180	0.67	0.0632	
NADH	0.00017	0.00026	0.00017	0.00016	0.00023	0.00013	-	-	-	-	0.00020	0.00013	0.66	-	
Ascorbic acid	0.00122	0.00272	0.00277	0.00129	0.00150	0.00114	0.00183	0.00177	0.00070	0.00078	0.00190	0.00124	0.66	0.1639	
Phosphorylcholine	0.00172	0.00222	0.00208	0.00184	0.00144	0.00090	0.00139	0.00106	0.00127	0.00141	0.00186	0.00120	0.65	0.0056	**
β-Ala	0.00609	0.00784	0.00776	0.00626	0.00685	0.00540	0.00489	0.00456	0.00280	0.00454	0.00696	0.00444	0.64	0.0024	**
β-Ala-Lys	0.00009	0.00016	0.00016	0.00012	0.00011	-	0.00011	0.00006	0.00008	-	0.00013	0.00008	0.63	0.0683	
1-Methylnicotinamide	0.00010	0.00015	0.00010	0.00013	0.00011	0.00008	0.00005	0.00009	0.00006	-	0.00012	0.00007	0.61	0.0091	**
Spermidine	0.00075	0.00109	0.00060	0.00088	0.00096	0.00047	0.00056	0.00039	0.00065	0.00048	0.00085	0.00051	0.60	0.0104	*
Glyceraldehyde 3-phosphate	0.00014	0.00017	0.00010	0.00020	0.00027	0.00011	0.00011	0.00010	0.00011	0.00010	0.00018	0.00010	0.59	0.0670	
Dihydroxyacetone phosphate	0.01359	0.01550	0.00708	0.01710	0.01828	0.00871	0.00758	0.00834	0.00736	0.00830	0.01431	0.00806	0.56	0.0332	*
Spermine	0.00018	0.00023	0.00016	0.00021	0.00022	0.00010	0.00008	0.00009	0.00016	0.00011	0.00020	0.00011	0.53	0.0010	**
N6-Methyllysine	0.00101	0.00130	0.00115	0.00149	0.00103	0.00074	0.00080	0.00043	0.00052	0.00063	0.00120	0.00063	0.52	0.0012	**
Fructose 1,6-diphosphate	0.02614	0.03829	0.00989	0.04208	0.03931	0.01199	0.01147	0.01157	0.00990	0.01264	0.03114	0.01151	0.37	0.0301	*
Hydroxyproline	0.02962	0.03298	0.03050	0.03077	0.02557	0.00988	0.01304	0.00902	0.00955	0.00942	0.02989	0.01018	0.34	0.0000	***
Taurocyamine	0.00058	0.00064	0.00082	0.00048	0.00067	0.00018	-	-	-	-	0.00064	0.00018	0.29	-	
2-(Creatinine-3-yl)propionic acid	-	-	-	0.00009	-	-	-	-	-	-	0.00009	-	not detected in Old	-	
6-Aminohexanoic acid	0.00017	0.00042	0.00032	0.00022	0.00027	-	-	-	-	-	0.00028	-	not detected in Old	-	
Cystathionine	-	-	0.00007	0.00010	-	-	-	-	-	-	0.00008	-	not detected in Old	-	
Isovalerylcarnitine	-	-	0.00019	-	-	-	-	-	-	-	0.00019	-	not detected in Old	-	
threo-β-Methylaspartic acid	-	-	-	0.00047	-	-	-	-	-	-	0.00047	-	not detected in Old	-	
XC0001	0.00007	0.00006	0.00004	-	0.00004	-	-	-	-	-	0.00005	-	not detected in Old	-	

Supplementary Table 2. List of gene expression of glycolytic pathway enzymes detected in microarray.

Gene	Accession no.	Description	Old vs Young
Hk1	NM_001146100	hexokinase 1 (Hk1), nuclear gene encoding mitochondrial protein	0.78
Hk2	NM_013820	hexokinase 2 (Hk2)	0.90
Gpi1	NM_008155	glucose phosphate isomerase 1 (Gpi1)	0.84
Pfkm	NM_021514	phosphofructokinase, muscle (Pfkm)	0.75
Aldoa	NM_001177307	aldolase A, fructose-bisphosphate (Aldoa)	0.88
Tpi1	NM_009415	triosephosphate isomerase 1 (Tpi1)	0.83
Gapdh	NM_008084	glyceraldehyde-3-phosphate dehydrogenase (Gapdh)	0.77
Pgk1	NM_008828	phosphoglycerate kinase 1 (Pgk1)	0.87
Pgam2	NM_018870	phosphoglycerate mutase 2 (Pgam2)	0.86
Eno3	NM_001136062	enolase 3, beta muscle (Eno3)	0.83
Pkm	NM_001253883	pyruvate kinase, muscle (Pkm)	0.94

Supplementary Table 3. Mouse-specific primer pairs used for quantitative real-time RT-PCR.

Gene	Forward	Reverse
36B4	GGCCCTGCACTCTCGCTTTC	TGCCAGGACGCGCTTGT
HK2	GGAACCCAGCTGTTTGACCA	CAGGGGAACGAGAAGGTGAAA
Pfkm	GCCATCGCCGTGTTGAC	GCCCTGACGGCAGCATT
Aldoa	GTGGGAAGAAGGAGAACCTG	CTGGAGTGTTGATGGAGCAG
PKM	TTGACTCTGCCCCCATCAC	GCAGGCCCAATGGTACAAAT
MyHC-Ⅰ	CCAAGGGCCTGAATGAGGAG	GCAAAGGCTCCAGGTCTGAG
MyHC-Ⅱa	AAGCGAAGAGTAAGGCTGTC	GTGATTGCTTGCAAAGGAAC
MyHC-Ⅱb	ACAAGCTGCGGGTGAAGAGC	CAGGACAGTGACAAAGAACG
Gpd1	GTGAGACGACCATCGGCTG	TTGGGTGTCTGCATCAGGT
Chkb	GCAAGACCCACGGACTACC	CAGGGAGTACCGACTGATCTC
Lypla2	AACACCATGTCTGTGCCCCT	CTGTGCCCTGTGTCTCCAAGT
Gpcpd1	ACATCGTGGTGCAGGGAACT	AAATGCTGCGCCATGACTG
Odc1	GCAGTCAACATCATTGCCAA	TGTTCATTTGACTCATCTTCATCGT
Amd2	TTTCACCGTGGCCTTCAGAT	GCCTCCTCCCTCTGGTCAGT
Smox	CACAGAGAGCTCCAAGACAGC	GGGCACTTGGATGGTAAAAG
Chrna1	GGTGTTCTACCTGCCCACAG	GCTCCACAATGACCAGAAGG
Ache	CAGCGCCACCGATACTCTG	CCTGCTTGCTATAGTGGTCGAAC
Col1a1	GACGCCATCAAGGTCTACTG	ACGGGAATCCATCGGTCA
Col3a1	GCCCACAGCCTTCTACAC	CCAGGGTCACCATTTCTC
Col6a2	GAACTTCCCTGCCAAACAGA	CACCTTGTGGAAGTTCTGCTC

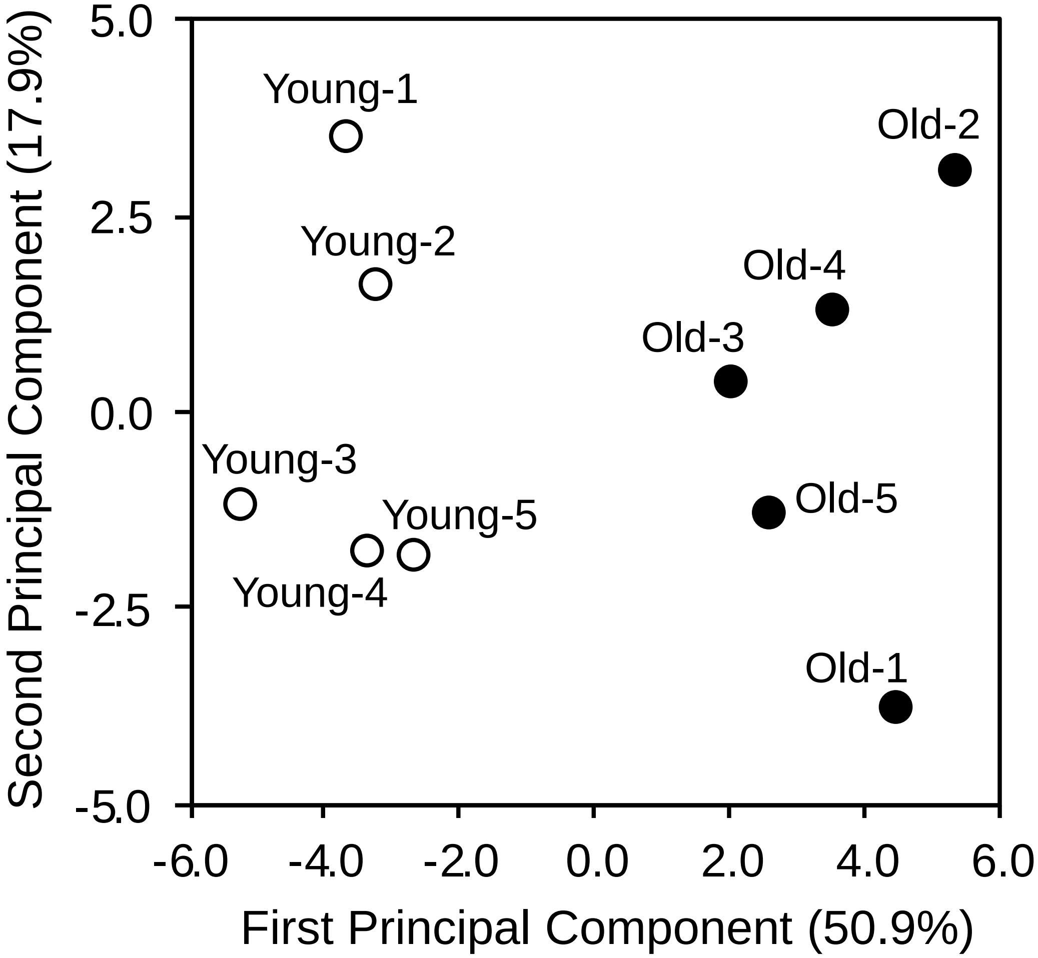

Supplementary Fig. 1. Plot of PCA of detected phosphatidylcholine (PC)/phosphatidylethanolamine (PE) peaks in lipid extracts from young and aged mice.

Five mice were used in each group (Young-1 to Young-5 for young mice and Old-1 to Old-5 for aged mice). The mice were same as used in Fig. 1. Plots of young (open circles) and aged mice (filled circles) are clearly distinguished on the first principal component axis (x-axis).

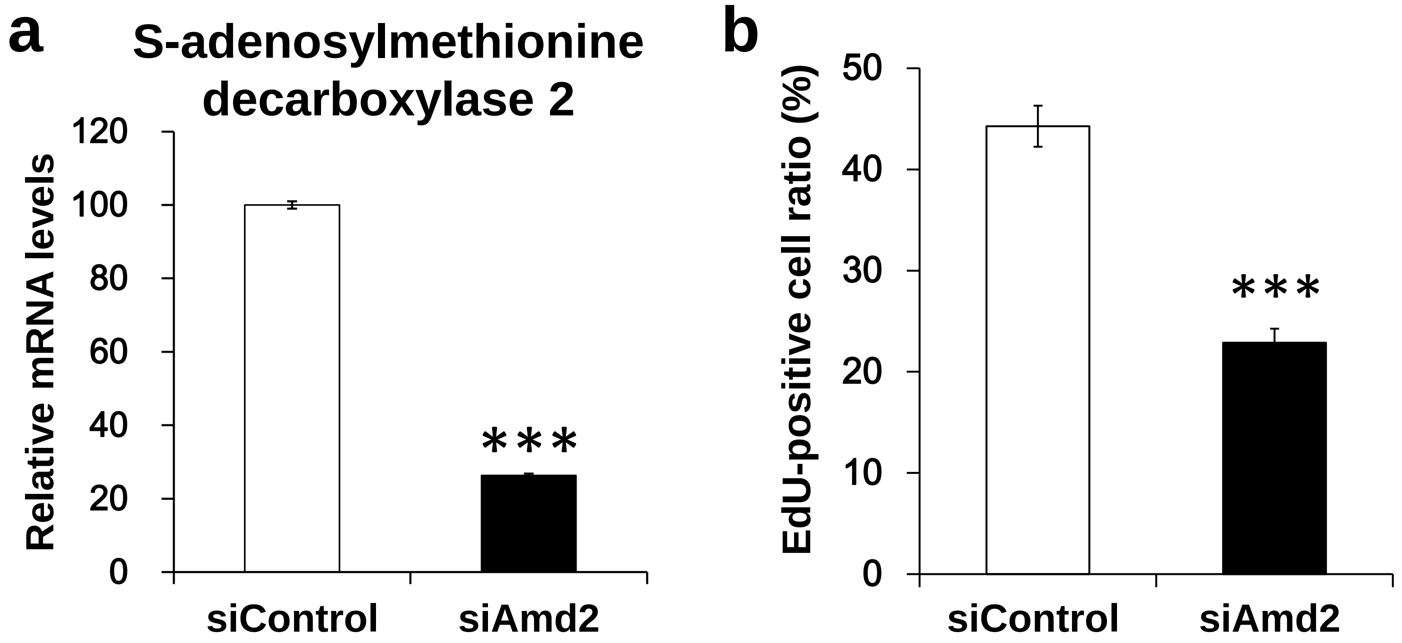

Supplementary Fig. 2. Knockdown of S-adenosylmethionine decarboxylase (Amd2) mRNA, and decreased cell proliferation of primary myoblasts.

a) siRNA successfully decreased Amd2 mRNA level. Data are expressed as mean ± SE (N = 4); ***$p < 0.001$.

b) Incorporation of EdU decreased following treatment of siRNA of Amd2. Data are expressed as mean ± SE (N = 8); ***$p < 0.001$.
