# Supplementary material for: Metabolomic Analysis of Skeletal Muscle in Aged Mice
Source: Sci Rep. 2019 Jul 18;9:10425. doi: 10.1038/s41598-019-46929-8 (PMC6639307; doi:10.1038/s41598-019-46929-8)
Supplement: Supplementary file 2 — Author signitures [file 41598_2019_46929_MOESM2_ESM.pdf]

nature publishing group npg

In accordance to Nature Publishing Groups Authorship Policy we agree to change the authors of the manuscript as indicated below.

NAME OF JOURNAL: Scientific Reports

TITLE OF MANUSCRIPT: **Metabolomic Analysis of Skeletal Muscle in Aged Mice**

MANUSCRIPT NUMBER: SREP-19-05029

CORRESPONDING AUTHORS NAME: Yasutomi Kamei

PREVIOUS AUTHOR NAMES:
Ran Uchitomi, Yukino Hatazawa, Takahiko Shimizu, Yasutomi Kamei

UPDATED AUTHOR NAMES:
Ran Uchitomi, Yukino Hatazawa, Nanami Senoo, Kiyoshi Yoshioka, Mariko Fujita, Takahiko Shimizu, Shinji Miura, Yusuke Ono, Yasutomi Kamei

CHANGE TO AUTHOR LIST: Nanami Senoo, Kiyoshi Yoshioka, Mariko Fujita, Shinji Miura, Yusuke Ono were added as authors.

Print Name	Signature	Date
Ran Uchitomi	Ran Uchitomi	June 10, 2019
Yukino Hatazawa	Yukino Hatazawa	June 10, 2019
Nanami Senoo	Nanami Senoo	June 10, 2019
Kiyoshi Yoshioka	Kiyoshi Yoshioka	June 10, 2019
Mariko Fujita	Mariko Fujita	June 10, 2019
Takahiko Shimizu	Takahiko Shimizu	June 10, 2019
Shinji Miura	Shinji Miura	June 10, 2019
Yusuke Ono	Yusuke Ono	June 10. 2019
Yasutomi Kamei	Yasutomi Kamei	June 10, 2019
